# Population Pharmacokinetics and Dosing Regimen of Lithium in Chinese Patients With Bipolar Disorder

**DOI:** 10.3389/fphar.2022.913935

**Published:** 2022-07-04

**Authors:** Zi-bin Jin, Zhuo Wu, Yi-fan Cui, Xue-peng Liu, Hong-bo Liang, Jia-yong You, Chen-yu Wang

**Affiliations:** ^1^ Department of Medical Psychology, The Affiliated Xuzhou Eastern Hospital of Xuzhou Medical University, Xuzhou, China; ^2^ Department of Pharmacy, Huashan Hospital, Fudan University, Shanghai, China; ^3^ Department of Pharmacy, Shanghai Chest Hospital, Shanghai Jiao Tong University, Shanghai, China; ^4^ School of Basic Medicine and Clinical Pharmacy, China Pharmaceutical University, Nanjing, China; ^5^ Department of Medical Service, Xuzhou Civil Affairs Psychiatric Hospital, Xuzhou, China

**Keywords:** lithium, bipolar disorder, population pharmacokinetics, Monte Carlo simulation, dosage regimen

## Abstract

**Background**: Lithium is an effective medication approved for the treatment of bipolar disorder (BD). It has a narrow therapeutic index (TI) and requires therapeutic drug monitoring. This study aimed to conduct a population pharmacokinetics (PPK) analysis of lithium and investigate the appropriateness of the dosing regimen according to different patient characteristics.

**Methods**: A total of 476 lithium concentrations from 268 patients with bipolar disorder were analyzed using nonlinear mixed-effects modeling. Monte Carlo simulations were employed to investigate the influence of covariates, such as weight, creatinine clearance, and daily doses of lithium concentrations, and to determine the individualized dosing regimens for patients.

**Results**: Lithium PK was described by a one-compartment model with first-order absorption and elimination processes. The typical estimated apparent clearance was 0.909 L/h^−1^ with 16.4% between-subject variability in the 62 kg patients with 116 ml/min creatinine clearance and 600 mg daily doses. To achieve a target trough concentration (0.4–0.8 mmol/L) in the maintenance phase, the regimen of 500 mg than 750 mg daily dose was recommended for patients with renal insufficiency and weighing 100 kg.

**Conclusion**: A PPK model for lithium was developed to determine the influence of patient characteristics on lithium pharmacokinetics. Weight, creatinine clearance, and total daily dose of lithium can affect the drug’s clearance. These results demonstrate the nonlinear renal excretion of lithium; hence, dosage adjustments are recommended for patients with renal insufficiency.

## 1 Introduction

Lithium is a first-line choice for the treatment of bipolar disorder (BD) for over 60 years (Malhi et al., 2017). Owing to the narrow therapeutic index of lithium and the therapeutic dose reaching drug toxicity, serum levels of lithium should be closely monitored during treatment (Yatham et al., 2018). Usually, 0.4 mmol/L is the minimum effective lithium concentration for bipolar disorder (Severus et al., 2008). Some patients experiencing acute manic phase episodes may need concentrations as high as 0.6–1.2 mmol/L. However, the range can be changed according to patients’ outcomes (Grandjean and Aubry, 2009).

After oral administration, lithium is completely absorbed in the upper gastrointestinal tract. *In vivo*, lithium ions do not bind to plasma proteins and are unevenly distributed throughout the body. In addition, lithium is not metabolized and is primarily excreted in the urine in its original form (Ward et al., 1994). Eighty percent of lithium is reabsorbed by passive diffusion at the renal tubules after it is filtered by the glomerulus, which may cause nonlinearity in the fractional excretion of lithium when the transporter is supersaturated (Price and Heninger, 1994).

With the rapid development of pharmacokinetics and computational modelling, population pharmacokinetics (PPK) has been widely used in the monitoring of clinical drug treatments and the optimization of personalized drug administration. Methaneethorn et al. investigated the influence of body weight and age on lithium clearance in a PPK analysis including 222 Thai patients with acute mania (Methaneethorn and Sringam, 2019). They reported that lithium clearance decreased as age increased in patients with equal body weight. In 52 children with intellectual disability aged 4–10 years old, Yuan et al. found that the lithium concentration over time was adequately described by a two-compartment model, with a transient absorption and first-order elimination process (Yuan et al., 2021). The inclusion of body weight as an allometric factor significantly improved the model fit, while age and sex were not associated with PKs of lithium. Yu et al. studied lithium carbonate PPK in 20 healthy young male Chinese volunteers; no covariate was retained in the final model because of the narrow distribution of demographic and biological variables (Yu et al., 2016). This limits the usefulness of this model in predicting the PK of lithium carbonate in the elderly. The effectiveness and accuracy of this model in predicting the PK of lithium carbonate in patients also needs to be further validated. To date, the PPK model is insufficient in the Chinese adult population with bipolar disorder, and the influence of disease physiological factors on the pharmacokinetics and pharmacodynamics of this population requires further investigation.

In this study, by using NONMEM and Monte Carlo, we attempted to establish a PPK model of lithium carbonate in patients with bipolar disorder, investigate the influence of covariates on drug concentration in the blood, and establish dosage recommendations for the personalized use of lithium carbonate in this population.

## 2 Materials and Methods

### 2.1 Population Pharmacokinetic Data

Chinese patients with bipolar disorder who received lithium carbonate between September 2016 and August 2021 at the Affiliated Xuzhou Eastern Hospital of Xuzhou Medical University were pooled to conduct this PPK analysis. The inclusion criteria were: 1) patients diagnosed with bipolar disorder; 2) patients on maintenance treatment of lithium; and 3) at least one corresponding lithium concentration sample. The exclusion criteria were as follows: 1) the use of any medication that might have clinically important interactions with lithium, such as diuretics, renin-angiotensin system antagonists, or serotonergic drugs (LITHIUM, 2020); and 2) a history of allergies or adverse reactions to lithium. This study was approved by the ethics committee of the Affiliated Xuzhou Eastern Hospital of Xuzhou Medical University (No. 20210928004). All the patients were exempt from informed consent for the research project as it does not involve personal privacy and commercial interests.

Serum lithium levels were measured before the morning dose and assessed using a phosphatase assay with the ADVIA 1800 clinical chemistry analyzer (Siemens Healthcare GmbH, Germany). The experimental procedures were performed in accordance with the manufacturer’s instructions. The calibration range of this method was 0.19–3.0 mmol/L. If the proportion of samples which are below the limit of quantification in the entire dataset is less than 10%, these samples were ignored. (Byon et al., 2013).

The following information was collected from each patient: age, weight (WT), sex, white blood cells (WBC), red blood cells (RBC), hematocrit (HCT), platelet count (PLT), albumin, alanine aminotransferase (ALT), aspartate aminotransferase (AST), blood urea nitrogen (BUN), total bilirubin (TBIL), serum creatinine (SCR), creatinine clearance (CRCL), and total daily dose (TDD). Referring to guidance in population modeling, if the missing covariates were less than 10%, the missing values were imputed using the median for continuous covariates (Byon et al., 2013).

### 2.2 Population Pharmacokinetic Analyses

Nonlinear mixed-effects modeling software (NONMEM, version 7.4.2, ICON Development Solutions, MD, United States) was used to develop a population PK model using first-order conditional estimation with interaction. NONMEM output was analyzed using Perl-speaks-NONMEM (PSN, version 4.7.0, Department of Pharmaceutical Biosciences, Uppsala University, Sweden) and R (version 3.4.1, R Foundation for Statistical Computing, Vienna, Austria).

### 2.2.1 Base Model

As only trough concentrations were collected in this population PK analysis, a one-compartment model with first-order absorption and elimination was used to describe the PK of lithium. The PK model was parameterized in terms of apparent clearance (CL/F) and apparent volume of distribution (V/F). The absorption rate constant (Ka) was fixed at 0.293 h^−1^ based on published data because no sampling was collected during the absorption phase.

An exponential model was chosen to describe between-subject variability (BSV) (Ahamadi et al., 2019):

$$P_{i}=P_{TV}\times e^{\left(\eta_{i}\right)}$$ (1)

where *P*_*i*_ represents the parameter of the *i*th individual, P_*tv*_ is the typical value of the population parameter, and *η*_*i*_ is the BSV for the *i*th individual with a mean of zero and variance of *ω* ^*2*^.

A proportional, additive or a combination model was used to describe the residual variability:

$$C_{i}=C_{pred}\times \left(1+\varepsilon_{1}\right)$$ (2)

$$C_{i}=C_{pred}+\varepsilon_{2}$$ (3)

$$C_{i}=C_{pred}\times \left(1+\varepsilon_{1}\right)+\varepsilon_{2}$$ (4)

where $C_{i}$ is the observed concentration of the *i*th individual, $C_{pred}$ is the individual predicted concentration, *ε*_*1*_ and *ε*_*2*_ are proportional and additive portions of residual variability. Both *ε*_*1*_ and *ε*_*2*_ are normally distributed with a mean of zero and variance σ^2^, respectively.

Based on the changes of object function value (OFV), parameter rationality, condition number, and plots of goodness-of-fit, base model selection was conducted (Donohue et al., 2011).

### 2.2.2 Covariate Model

The covariate analysis was conducted using a three-step approach (Byon et al., 2013). In the first step, a graphical display of the correlation between the random effects of PK parameters and covariates was evaluated to explore the sources of variability. Only covariates of interest were used in the next step. Secondly, stepwise forward inclusion selection was evaluated. Statistically significant covariates were determined with a decrease of OFV by > 3.84 (*p* < 0.05). Finally, a stepwise backward elimination process was initiated. The covariate was temporarily removed with an increase of OFV by < 6.63 (*p* > 0.01).

Relationships between potential covariates and parameters were explored using linear additive and power functions for the continuous covariates (Eqs 5, 6), and categorical covariates (Eq. 7) (Mandema et al., 1992):

$$P_{i}=P_{TV}\times \left(1+\theta \times \frac{Cov_{i}}{Cov_{median}}\right)$$ (5)

$$P_{i}=P_{TV}\times {\left(\frac{Cov_{i}}{Cov_{median}}\right)}^{\theta }$$ (6)

$$P_{i}=P_{TV}\times \left(1+\theta^{Cov_{i}}\right)$$ (7)

where *P*_*i*_ represents the parameter for the *i*th individual, $P_{TV}$ is the typical value of the parameter, *Cov*_*i*_ is the covariate value of the *i*th individual, *Cov*_*median*_ is the median value of the continuous covariate and *θ* is the coefficient term of the covariate effect to be estimated.

### 2.3 Model Evaluation

Goodness-of-fit plots were used to evaluate the fitness of the final model to the data. Scatterplots were used to evaluate observed concentration (DV) versus population predicted concentration (PRED), DV versus individual predicted concentrations (IPRED), conditional weighted residuals (CWRES) versus PRED and CWRES versus time.

The model was also evaluated internally using a bootstrap analysis (Ette et al., 2003). During the bootstrap process, each parameter was evaluated repeatedly by applying the final model to 1,000 bootstrapped datasets. The 2.5th, 50th, and 97.5th percentiles of the population PK parameter values from bootstrap datasets were compared with those from the final model.

To evaluate the predictive performance of the final model, a visual predictive check (VPC) was stratified to compare the observed concentrations and model predictions. The VPC approach was conducted by simulating 1,000 datasets from the final model and comparing the observed data with the simulated data.

### 2.4 Model-Based Simulation

Monte Carlo simulations were performed to predict the trough concentration after 7-day multiple oral doses of different dosing regimens based on the final population PK model. The daily dose of lithium carbonate was simulated from 250 to 1,000 mg. One thousand simulations were performed using the initial dataset, and the steady-state trough concentrations of each simulated subject were calculated.

## 3 Results

### 3.1 Demographics

A total of 476 plasma lithium measurements obtained from 268 patients were used for pharmacokinetic analysis. The baseline demographic and general clinical information of the patients used for model building is summarized in Table 1. The proportion of women and men in this study was 66.8 and 33.2%, respectively. The median age was 31.0 (13.0–77.0) years, and 89.9% of them were adults (age > 16 years). The median weight and daily lithium dose used in the population were 62.0 (35.0–110) kg and 600 (150–1,500) mg, respectively. The mean creatinine clearance rate was 118 ml/min, ranging from 61.7 to 226 ml/min.

**TABLE 1:** Demographic and characteristics of patients.

Covariate	Median (min-max)	Mean ± SD
Number of patients	268 (100%)	
Number of PK Samples	476 (100%)	
Sex		
Male (%)	89 (33.2%)	
Female (%)	179 (66.8%)	
Age group		
Adult (%)	241 (89.9%)	
Child (%)	27 (10.1%)	
Dosage form		
Ordinary tablet	64 (23.9%)	
Sustained release tablet	204 (76.1%)	
Age (years)	31.0 (13.0–77.0)	35.0 ± 14.5
Weight (kg)	62.0 (35.0–110)	63.7 ± 11.1
Total daily dose (mg)	600 (150–1,500)	720 ± 236
WBC (10^9^/L)	7.03 (2.86–16.5)	7.32 ± 2.25
RBC (10^12^/L)	4.43 (2.88–10.2)	4.52 ± 0.734
Hematocrit (%)	40.7 (29.5–52.8)	40.7 ± 4.7
PLT (10^9^/L)	232 (60.0–734)	236 ± 70.8
Albumin (g/L)	41.9 (33.3–68.0)	42.2 ± 3.66
TBIL (μmol/L)	9.40 (2.62–34.5)	10.3 ± 4.9
AST (U/L)	19.0 (6.00–303)	23.4 ± 20.5
ALT (U/L)	20.0 (5.00–298)	27.1 ± 27.5
BUN (mmol/L)	3.88 (1.29–13.5)	4.09 ± 1.35
SCR (μmol/L)	62.0 (40.0–115)	63.7 ± 12.6
CRCL (ml/min)*	116 (61.7–226)	118 ± 30.3

* CRCL = [(140-Age) × weight (kg)]/[0.818×Scr(μmol/L)] × k, where k is 1 for male and 0.85 for female.

ALT, alanine aminotransferase; AST, aspartate aminotransferase; BUN, blood urea nitrogen; CRCL, creatinine clearance rate; PLT, platelet count; RBC, red blood cell; SCR, serum creatinine; TBIL, total bilirubin; WBC, white blood cells.

### 3.2 Population Pharmacokinetic Model

The random effects of CL/F were significantly correlated with weight, creatinine clearance, daily lithium dose, and sex by analyzing the correlation diagram. Therefore, the influence of these covariates on the CL/F ratio was tested using a stepwise approach. We observed a statistically significant drop in OFV (70.12) during the covariate screening of daily dose. The decrease in OFV was also greater than 3.84 after we included weight and creatinine clearance in the final model. However, the addition of sex did not meet the criteria for statistical significance (*p* < 0.05). No covariates were removed during backward elimination (*p* < 0.01). The final pharmacokinetic parameters of lithium are listed in Table 2 and Eqs 8–10:

$$\text{CL}/\text{F}\left(\text{L}/\text{h}\right)=0.909\times {\left(\text{TDD}/600\right)}^{0.354}\times {\left(\text{WT}/62\right)}^{0.33}\times {\left(\text{CRCL}/116\right)}^{0.186}\times {\text{e}}^{0.027}$$ (8)

$$\text{V}/\text{F}\left(\text{L}\right)=10.9\times {\text{e}}^{0.162}$$ (9)

$$\text{Ka}\left({\text{h}}^{-1}\right)=0.293\;\left(\text{FIX}\right)$$ (10)

**TABLE 2:** Population-pharmacokinetic parameter estimates and bootstrap evaluation.

Parameters	Base model		Final model		
	Parameter estimates (RSE%)	Shrinkage (%)	Parameter estimates (RSE%)	Shrinkage (%)	Bootstrap median (2.5%–97.5%)
CL (L/h)	0.969 (4)		0.909 (3)		0.906 (0.855–0.954)
V(L)	9.27 (16)		10.9 (12)		10.8 (8.0–13.4)
Ka (h^−1^)	0.293 [fixed]		0.293 [fixed]		0.293 [fixed]
WT on CL	—		0.33 (29)		0.330 (0.134–0.520)
CRCL on CL	—		0.186 (29)		0.184 (0.075–0.291)
TDD on CL	—		0.354 (12)		0.351 (0.267–0.435)
Between subject variability					
CL (%)	20.8 (9)	24	16.4 (10)	30	16.1 (12.8–19.5)
V(%)	40.4 (21)	63	40.2 (20)	62	39.4 (16.0–52.6)
Residual variability					
Additive error (mmol/L)	0.0236 (14)	22	0.0218 (13)	20	0.0216 (0.0166–0.0274)

#### 3.3 Model Evaluation

**FIGURE 1:**
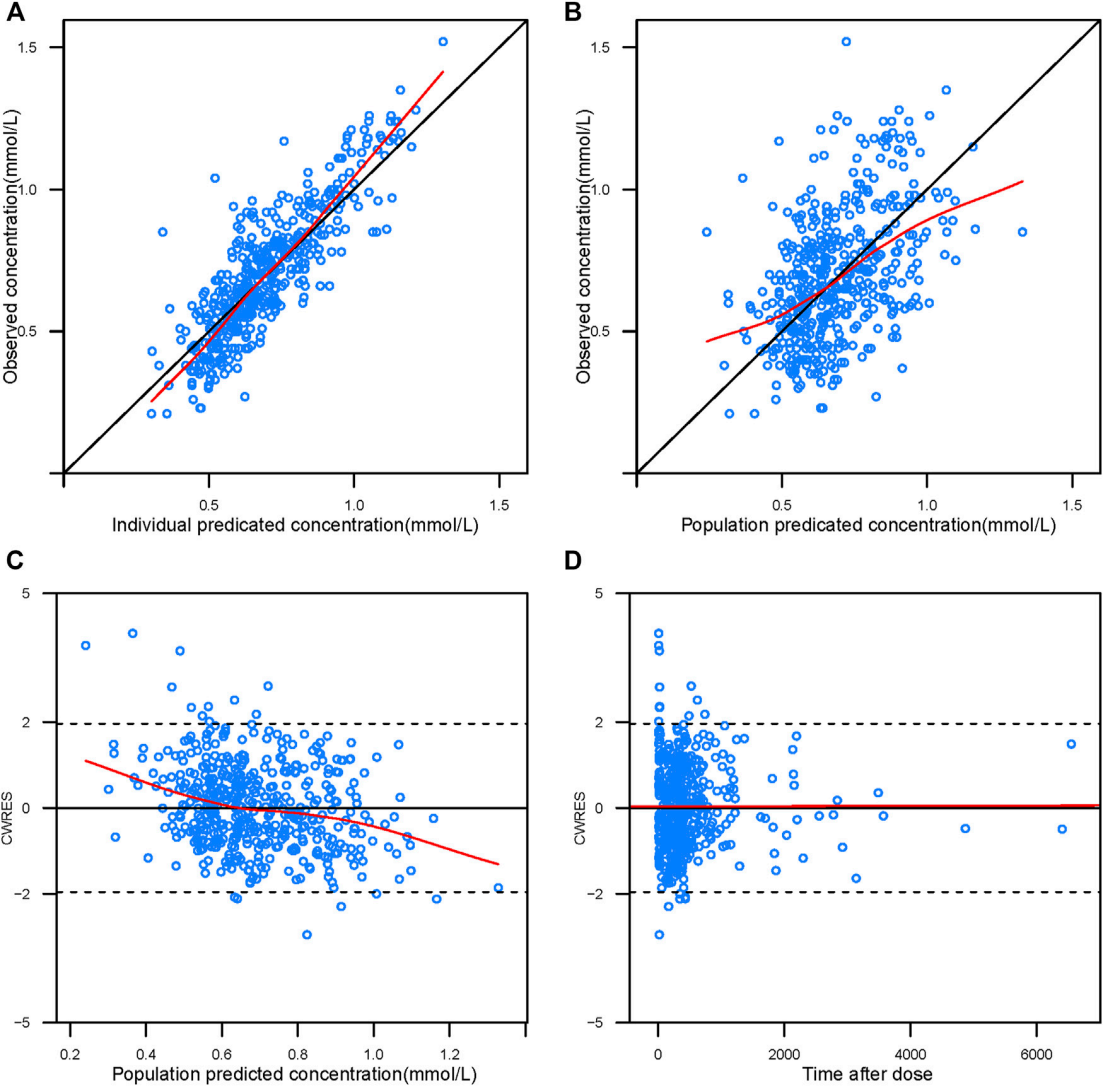
Goodness-of-fit plots of the final population-pharmacokinetic model. The upper left plot represents the observations versus the population predictions **(A)**. The upper right plot represents the observations versus the individual predictions **(B)**. The lower left plot represents the conditional weighted residuals versus the population predictions **(C)**. The lower right plot represents the conditional weighted residuals versus the time after dose **(D)**. The red line represents the locally weighted scatterplot smoothing line.

The goodness-of-fit plots of the final model are shown in Figure 1. The population and individual prediction values of the final model were evenly distributed on both sides of the reference line (Figures 1A, B). CWRES plots illustrating individual predicted concentrations and time after dose are randomly scattered with most CWRES ranging from −2 to +2, and the trend line is close to zero (Figures 1C, D). In other words, all figures illustrate the good predictive performance of the proposed model.

Additionally, one thousand bootstrap datasets generated by resampling from the original dataset were repeatedly fitted with the final model to estimate the model parameters, yielding 96.5% successful convergence. The estimates from the original models were within the 95% CIs of the bootstrap estimates (Table 2), confirming the robustness of the model.

**FIGURE 2:**
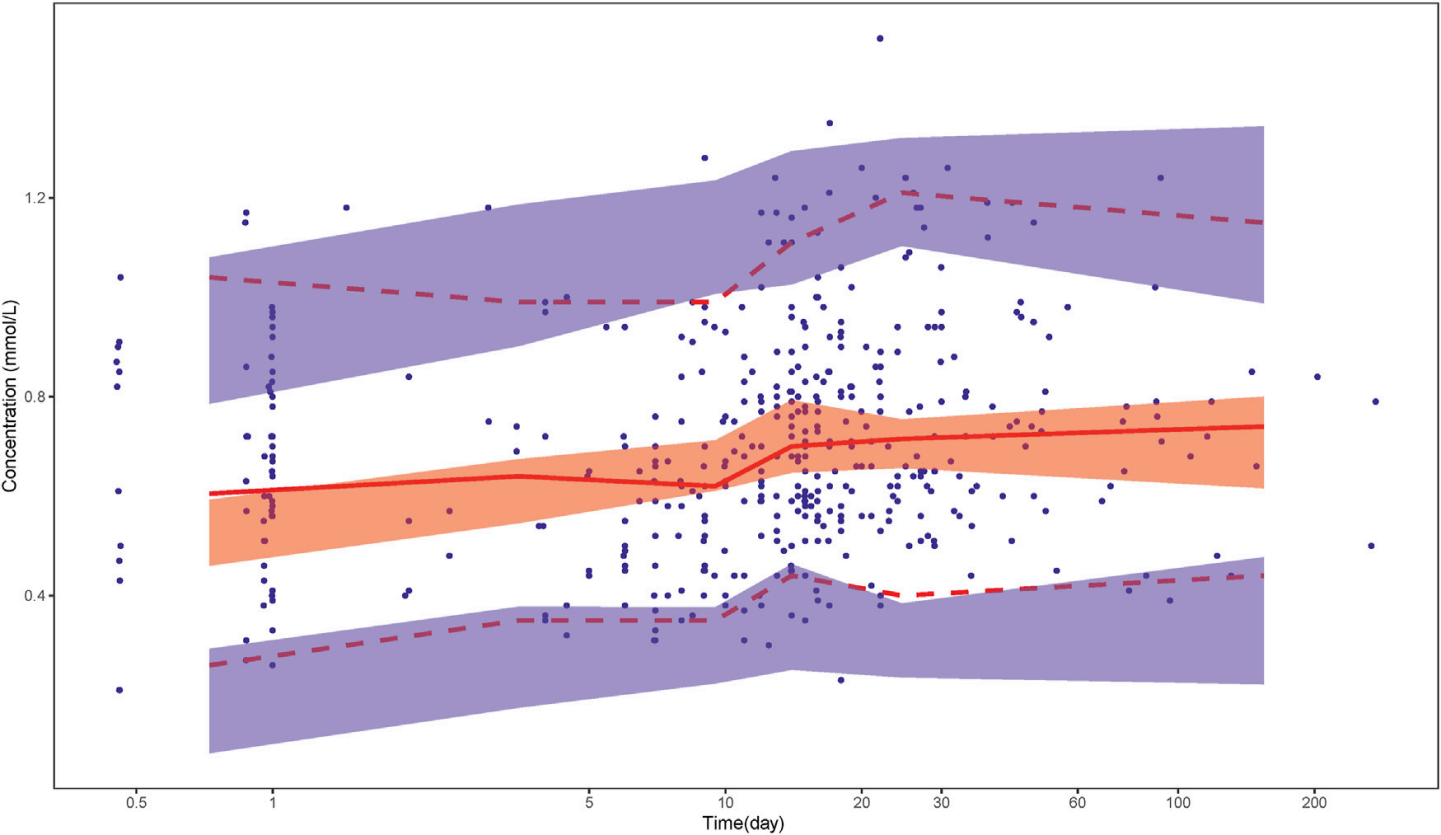
Visual predictive check. Solid dots represent observed data. Lines represent the 5% (dashed), 50% (solid), and 95% (dashed) percentiles of the observed data. Shaded areas represent nonparametric 95% confidence intervals about the 5% (light blue), 50% (light red), and 95% (light blue) percentiles of the predicted concentrations.

The VPC results (Figure 2) show that the observed plasma concentration data mostly fits within the 95% confidence intervals of the 5th, 50th, and 95th percentiles of the simulated data, which indicates that the final pharmacokinetic model can adequately describe the observed concentrations.

We performed a sensitivity analysis to determine the effect of fixed Ka on the final model by varying the Ka values within the range of 0.146–0.586 h^−1^, and the estimated value of CL/F was slightly changed between 0.825 and 0.959 L/h.

### 3.4 Model-Based Simulation

**FIGURE 3:**
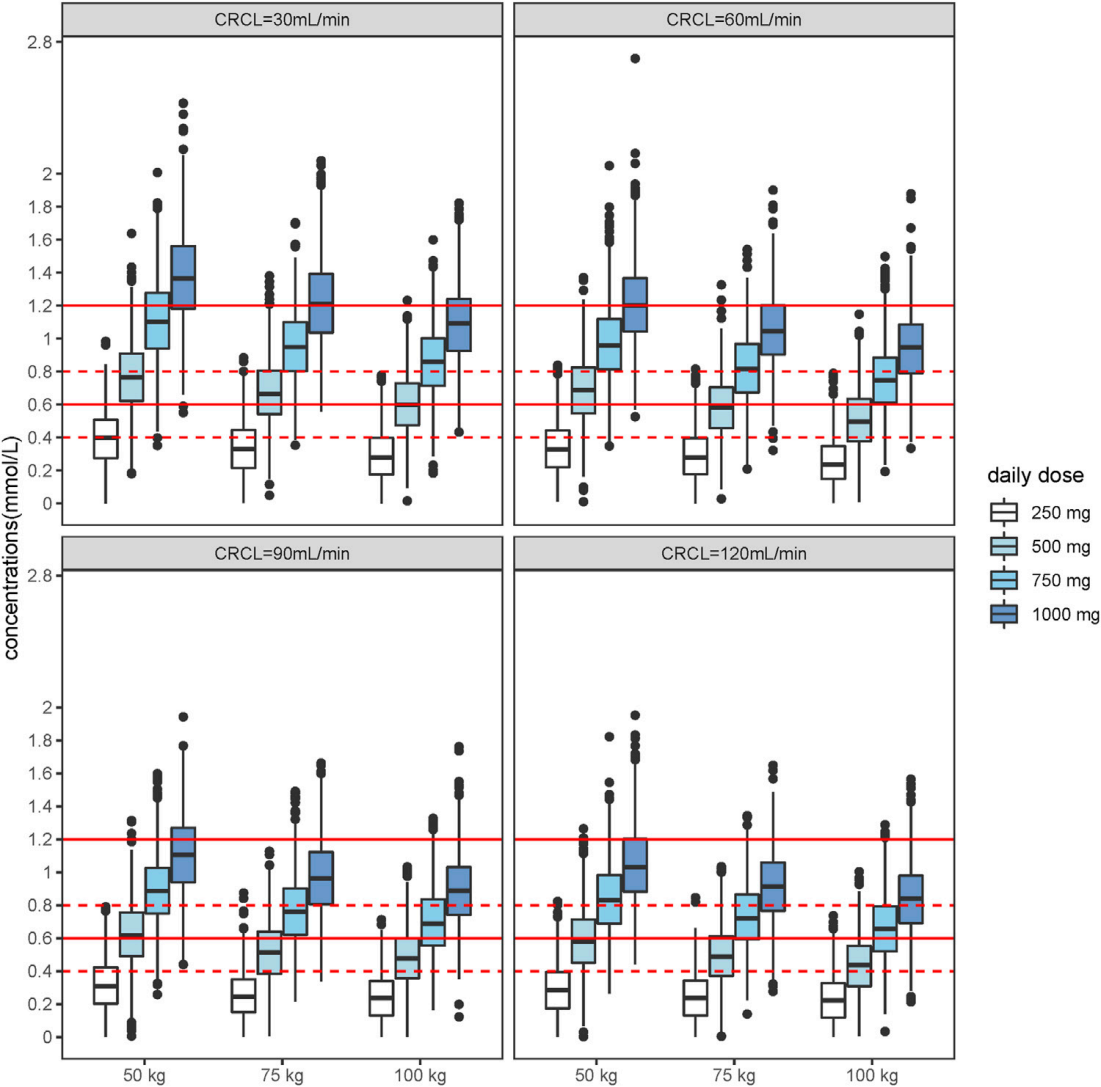
Boxplot of the distributions of simulated steady state trough lithium concentrations for patients with different weight and CRCL levels. Patients with different weight levels from 50, 75, 100 kg, and CRCL levels from 30 ml/min, 60 ml/min, 90 ml/min, 120 ml/min were set for the simulation. Dashed horizontal lines represent the therapeutic target range (0.6–1.2 mmol/L) for the acute phase and point horizontal lines represent the therapeutic target range (04–0.8 mmol/L) for maintenance phase.

The lithium steady-state trough concentrations of 1,000 individuals were simulated under different scenarios, including significant covariates based on the final model. The ranges we set were 50–100 kg for weight, 250–1,000 mg for lithium daily dose, and creatinine clearance rate between 30 and 120 ml/min. The simulation results are shown in Figure 3 **.**

Appropriate dosage regimens meeting the target lithium concentrations were selected for individualized administration according to the simulation results. During the maintenance period, a daily dose of 750 mg is recommended for patients weighing 100 kg with normal renal function (CrCL = 120 ml/min). The CL/F of patients would decrease by 23% when their renal function is poor (CrCL = 30 ml/min), presenting a 500 mg daily dose as a better choice for maintenance. Patients need higher doses in the acute phase, and the concentration of lithium is in the effective treatment window of 1,000 mg daily for 100 kg patients with normal renal function. However, a daily dose of 750 mg is recommended for patients with poor renal function during the acute period.

## 4 Discussion

To the best of our knowledge, this is the first study to report a population PK model of lithium in Chinese patients with bipolar disorder. The PK of lithium was characterized using a one-compartment model with first-order absorption and elimination. WT, CRCL, and TDD were identified as the CL/F covariates. Our study showed that for a typical 62 kg patient with a CRCL of 116 ml/min receiving a daily lithium dose of 600 mg, the typical CL/F value was estimated to be 0.909 L/h, which is consistent with previous reported clearance between 0.51 to 1.47 L/h (Wing et al., 1997; ElDesoky et al., 2008; Findling et al., 2010; Landersdorfer et al., 2017).

In this population PK study, we found that TDD could be incorporated into the final model as a covariate, indicating nonlinear clearance of the drug in patients with bipolar disorder. In the PPK model conducted by Yuan et al., TDD was also evaluated as a covariate. However, it was not found that TDD could significantly influence the PK parameter of lithium, which may be because only 170 lithium plasma concentrations were included in the analysis (Yuan et al., 2021). In our study, a total number of 476 concentrations were included, which is the largest of all previously reported population PK studies. The CL/F ratio of lithium increased nonlinearly with TDD, which is consistent with the results of Uwai et al. They reported that the infusion of an inhibitor of sodium-phosphate cotransporter decreased the fractional reabsorption of lithium in rats administered 2.5 mg/kg of lithium chloride but did not affect it in rats administered 25 mg/kg (Uwai et al., 2018). These results demonstrate the nonlinearity of renal excretion of lithium. Further research with a larger sample size and intensive sampling will provide a more specific picture of the nonlinear behavior of lithium kinetics.

And the impact of CRCL and WT on the PK of lithium has been previously reported (Jermain et al., 1991; ElDesoky et al., 2008; Perez-Castello et al., 2016; Alqahtani et al., 2020). This is an expected result, as lithium is mainly cleared via the kidney. Lithium is freely filtered through the glomeruli, and protein binding of lithium in the plasma is negligible. In our study, when patients had severe renal insufficiency (CRCL = 30 ml/min), the typical CL/F of lithium decreased by 23%, which was consistent with the results of the Alqahtani group (Alqahtani et al., 2020). Their study in patients with bipolar disorder in Saudi Arabia suggested that the 15% lowering of CL, was attributed to the reduced renal clearance from 120 to 30 ml/min. Similarly, as body weight increases, renal blood flow and lithium clearance decrease. As the weight levels increased from 50 to 100 kg, the CL increased by 28% from 0.83 L/h 1.06 L/h.

To guide therapeutic dosing of lithium, we added a simulation based on the PK parameters. The acute treatment concentration range is 0.6–1.2 mmol/L for Chinese patients, and the maintenance treatment concentration range is 0.4–0.8 mmol/L. Several conclusions can be drawn from this study. First, lithium concentration can be maintained in the range of 0.4–0.8 mmol/L for 500–750 mg daily and the range of 0.6–1.2 mmol/L for 750–1,000 mg daily in patients with normal renal function. Second, the lithium concentration can be maintained in the range of 0.4–0.8 mmol/L for 500 mg daily and in the range of 0.6–1.2 mmol/L for 500–750 mg daily in patients with poor renal function.

This study had some limitations. First, owing to the retrospective nature of this study, all information may not have been properly controlled. For example, all available concentrations were collected as trough, the Ka parameter was fixed, and Vd/F couldn’t be a reliable estimate. Secondly, all samples were from the same hospital. More validations of this model required samples from other hospitals.

## Conclusion

In summary, we developed a population PK model of lithium in patients with bipolar disorder. Based on the model simulation results, the optimal strategy depends on renal function and weight. We also demonstrated the nonlinearity of the renal excretion of lithium, but further research is required.

## Data Availability

The raw data supporting the conclusion of this article will be made available by the authors, without undue reservation.
