# Supplementary material for: Population Pharmacokinetics and Dosing Regimen of Lithium in Chinese Patients With Bipolar Disorder
Source: Front Pharmacol. 2022 Jul 4;13:913935. doi: 10.3389/fphar.2022.913935 (PMC9289112; doi:10.3389/fphar.2022.913935)
Supplement: Supplementary file 1 [file Table1.DOCX]

| **Covariate** | **Median (Min-Max)** | **Mean±SD** |
| --- | --- | --- |
| Number of patients | 268 (100%) |  |
| Number of PK Samples | 476 (100%) |  |
| SEX |  |  |
| Male (%) | 89 (33.2%) |  |
| Female (%) | 179 (66.8%) |  |
| Age group |  |  |
| Adult (%) | 241 (89.9%) |  |
| Child (%) | 27 (10.1%) |  |
| Dosage form |  |  |
| Ordinary tablet | 64 (23.9%) |  |
| Sustained release tablet | 204 (76.1%) |  |
| Age (years) | 31.0 (13.0-77.0) | 35.0±14.5 |
| Weight (kg) | 62.0 (35.0- 110) | 63.7±11.1 |
| Total daily dose (mg) | 600 (150-1500) | 720±236 |
| WBC (10^9^/L) | 7.03 (2.86-16.5) | 7.32±2.25 |
| RBC (10^12^/L) | 4.43 (2.88-10.2) | 4.52±0.734 |
| Hematocrit (%) | 40.7(29.5-52.8) | 40.7±4.7 |
| PLT (10^9^/L) | 232 (60.0-734) | 236±70.8 |
| Albumin (g/L) | 41.9 (33.3-68.0) | 42.2±3.66 |
| TBIL (μmol/L) | 9.40 (2.62-34.5) | 10.3±4.9 |
| AST (U/L) | 19.0 (6.00-303) | 23.4±20.5 |
| ALT (U/L) | 20.0 (5.00-298) | 27.1±27.5 |
| BUN (mmol/L) | 3.88 (1.29-13.5) | 4.09±1.35 |
| SCR (μmol/L) | 62.0 (40.0-115) | 63.7±12.6 |
| CRCL (mL/min)* | 116(61.7-226) | 118±30.3 |

**Table 1**. Demographic and characteristics of patients

ALT, Alanine aminotransferase; AST, Aspartate aminotransferase; BUN, Blood urea nitrogen; CRCL, Creatinine clearance rate; PLT, Platelet count; RBC, Red blood cell; SCR, Serum creatinine; TBIL, Total bilirubin; WBC, White blood cells.

* CRCL=[(140-Age)×weight(kg)]/[0.818×Scr(μmol/L)] × k, where k is 1 for male and 0.85 for female.
