# Supplementary material for: Population Pharmacokinetics and Dosing Regimen of Lithium in Chinese Patients With Bipolar Disorder
Source: Front Pharmacol. 2022 Jul 4;13:913935. doi: 10.3389/fphar.2022.913935 (PMC9289112; doi:10.3389/fphar.2022.913935)
Supplement: Supplementary file 2 [file Table2.DOCX]

| **Parameters** | **Base model** |  | **Final model** | | |
| --- | --- | --- | --- | --- | --- |
|  | Parameter estimates  (RSE%) | Shrinkage  (%) | Parameter estimates  (RSE%) | Shrinkage  (%) | Bootstrap  Median (2.5% - 97.5%) |
| CL (L/h) | 0.969 (4) |  | 0.909 (3) |  | 0.906 (0.855-0.954) |
| V(L) | 9.27 (16) |  | 10.9 (12) |  | 10.8 (8.0-13.4) |
| Ka (h^-1^) | 0.293 [fixed] |  | 0.293 [fixed] |  | 0.293 [fixed] |
| WT on CL | / |  | 0.33 (29) |  | 0.330 (0.134-0.520) |
| CRCL on CL | / |  | 0.186 (29) |  | 0.184 (0.075-0.291) |
| DD on CL | / |  | 0.354 (12) |  | 0.351 (0.267-0.435) |
| Between subject variability |  |  |  |  |  |
| CL (%) | 20.8 (9) | 24 | 16.4 (10) | 30 | 16.1 (12.8-19.5) |
| V(%) | 40.4 (21) | 63 | 40.2 (20) | 62 | 39.4 (16.0-52.6) |
| Residual variability |  |  |  |  |  |
| additive error (mmol/L) | 0.0236 (14) | 22 | 0.0218 (13) | 20 | 0.0216 (0.0166-0.0274) |

**Table 2**. Population-pharmacokinetic parameter estimates and bootstrap evaluation
